# Supplementary material for: Dissecting the Therapeutic Mechanisms of Sphingosine-1-Phosphate Receptor Agonism during Ischaemia and Reperfusion
Source: Int J Mol Sci. 2023 Jul 7;24(13):11192. doi: 10.3390/ijms241311192 (PMC10342646; doi:10.3390/ijms241311192)
Supplement: Supplementary file 1 [file ijms-24-11192-s001.zip › ijms-2421529-supplementary.pdf]

Article

Dissecting the Therapeutic Mechanisms of Sphingosine-1-Phosphate Receptor Agonism during Ischaemia-Reperfusion Injury

Georgina C. Wilkins †, Jenny Gilmour †, Eirini Giannoudaki, John A. Kirby, Neil S. Sheerin * and Simi Ali *

Theme: Immunity and Inflammation, Translational and Clinical Research Institute, Faculty of Medical Sciences, Newcastle University, Newcastle upon Tyne NE24HH, UK; georgie.wilkins@newcastle.ac.uk (G.C.W.); jenny.gilmour@newcastle.ac.uk (J.G.); giannoue@tcd.ie (E.G.); john.kirby@newcastle.ac.uk (J.A.K.)

* Correspondence: neil.sheerin@newcastle.ac.uk (N.S.S.); simi.ali@newcastle.ac.uk (S.A.);
Tel.: +44-(0)-19120-87158

† These authors contributed equally to this work.

Supplementary Materials:

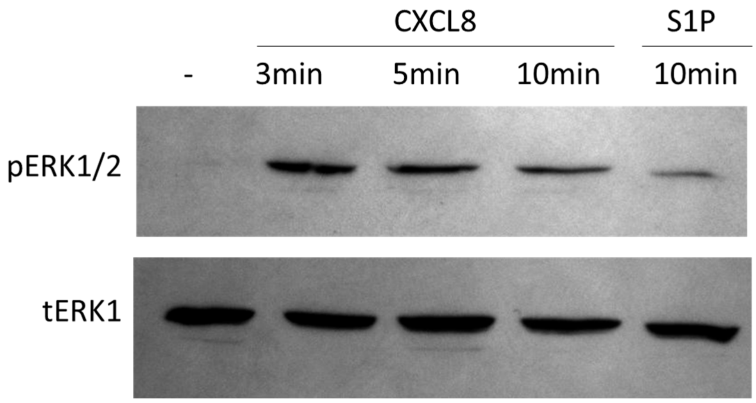

Supplementary figure 1. S1P receptor signalling in neutrophils. Neutrophils were isolated from whole human blood and used immediately in experiments. Effect of CXCL8 on pERK expression in neutrophils. Isolated neutrophils were treated with 100 ng/mL CXCL8 for between 3 and 10 minutes or with1 μM S1P for 10 minutes. Neutrophils were lysed and used in western blotting experiments with pERK1/2 and total ERK as a loading control. Data are representative of 5 independent experiments.

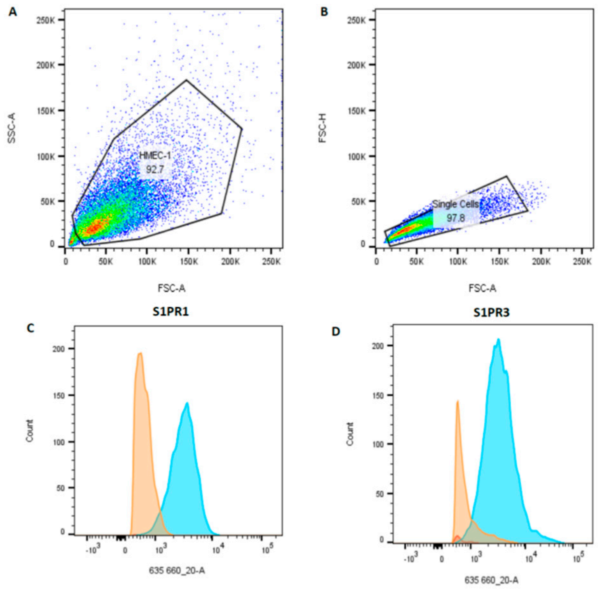

Supplementary figure 2. FACs quantification of S1PR1 and S1PR3 protein expression in endothelial cells. HMEC-1 were grown to confluence in T75 flasks, then S1PR1 and S1PR3 protein expression was quantified using FACs. (A) Cells were gated, identified as a large and non-granular population. (B) The single cell population of cells were identified. (C) S1PR1 expression. (D) S1PR3 expression. Red= unstained. Orange = IgG. Blue = S1PR1/S1PR3. Data representative of three independent experiments.

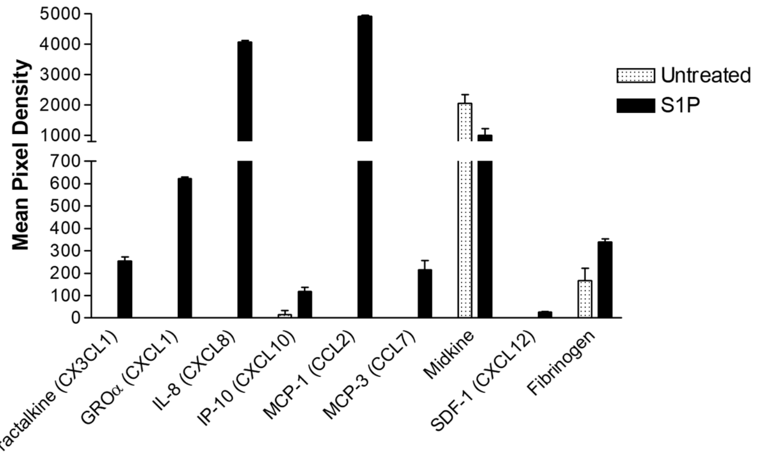

Supplementary figure 3. Chemokine array using HUVECs. HUVEC were treated with or without 10μM S1P for 24 hours. Supernatants were collected for use in a chemokine array. Diagram shows mean pixel densities for different chemokines in the array; bars represent means ± SEM. N=2.

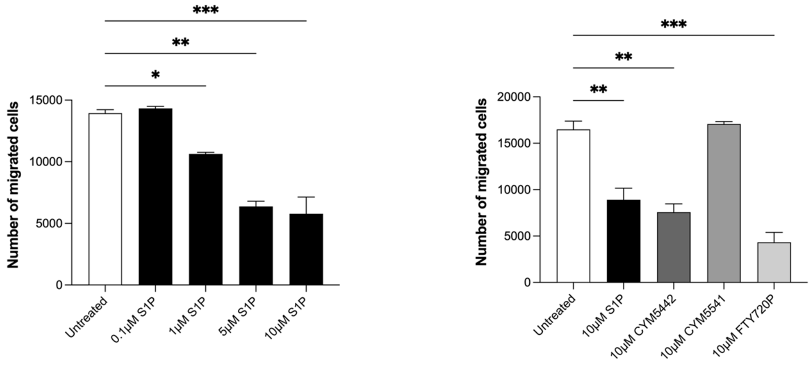

Supplementary figure 4. S1P and associated agonist treatment reduces neutrophil trans-endothelial migration towards CXCL8 using HMEC-1 (A) HMEC-1 were seeded on chemotaxis filters and treated overnight with 0.1-10μM S1P. Neutrophils isolated from whole human blood were added to the top chamber of the chemotaxis filter and left to migrate for 2 hours towards 10ng/mL CXCL8. Total migrated cells were counted by flow cytometry. Data representative of 4 independent experiments. (B) HMEC-1 were seeded on chemotaxis filters and treated overnight with 10μM S1P, CYM5442, CYM5541 or FTY720P. Neutrophils isolated from whole human blood were added to the top chamber of the chemotaxis filter and left to migrate for 2 hours towards 10ng/mL CXCL8. Total migrated cells were counted by flow cytometry. Data representative of 4 independent experiments. Statistical significance determined by a one-way ANOVA.
